# Exploring the impact of choral singing on older adult community health and well-being

**DOI:** 10.1093/geroni/igaf122.3585

**Published:** 2025-12-31

**Authors:** Noell Rowan, Sandra Quinn, Mary Ann East, Meg Jordan, Marieh Arnett

**Affiliations:** University of North Carolina Wilmington, Wilmington, North Carolina, United States; University of Maryland, College Park, College Park, Maryland, United States; Encore Creativity for Older Adults, Washington D.C., District of Columbia, United States; University of Maryland, College Park, College Park, Maryland, United States; University of Maryland, College Park, College Park, Maryland, United States

## Abstract

There is increasing interest in community engaged research on the impact of singing on health and well-being of older adults. As its aim, Community-based participatory research (CBPR) blends knowledge with action to create positive and lasting change. Encore Creativity for Older Adults (hereafter Encore) is the largest choral program for older adults in the United States. Community members within Encore were engaged as members of the research team as advisors to the long-term planning process. This cross-sectional survey explored the musical/singing experience, physical/mental health, social connection, loneliness and well-being of singers. Of the (N = 886) participants, over 41% have been with Encore for four years or more, including 15% with more than 10 years. Key findings included 73% reported their health as very good/excellent, with a mean of 4.11 (out of 5) on mental health. Statistically significant differences were noted in life satisfaction with those newest to Encore reporting lower life satisfaction than those with 1 year or more Encore experience. Significant predictors of current health were mental health, health limitations, and life satisfaction. Encore experience became a significant predictor when it interacted with mental health. Singers reported that having a sense of purpose, life-long learning, health and social benefits, social connection, and ease of participation kept them engaged with Encore. There were no significant differences in loneliness by years of Encore experience, suggesting protective benefits across all stages of participation. Implications include practice skills and advocacy to increase music engagement and the potential impact on health/mental health and overall well-being.

